# Differences in temporal order memory among young, middle-aged, and older adults may depend on the level of interference

**DOI:** 10.3389/fnagi.2015.00028

**Published:** 2015-03-18

**Authors:** Lindsay J. Rotblatt, Catherine A. Sumida, Emily J. Van Etten, Eva Pirogovsky Turk, Jerlyn C. Tolentino, Paul E. Gilbert

**Affiliations:** ^1^Department of Psychology, San Diego State UniversitySan Diego, CA, USA; ^2^Department of Psychiatry, University of California San DiegoLa Jolla, CA, USA; ^3^Veterans Affairs San Diego Health Care System, Research ServiceSan Diego, CA, USA; ^4^San Diego State University—University of California San Diego Joint Doctoral Program in Clinical PsychologySan Diego, CA, USA

**Keywords:** temporal order, interference, aging, middle age, pattern separation

## Abstract

Age-related changes in temporal order memory have been well documented in older adults; however, little is known about this ability during middle age. We tested healthy young, middle-aged, and older adults on a previously published visuospatial temporal order memory test involving high and low interference conditions. When interference was low, young and middle-aged adults did not differ, but both groups significantly outperformed older adults. However, when interference was high, significant differences were found among all three age groups. The data provide evidence that temporal order memory may begin to decline in middle age, particularly when temporal interference is high.

## Introduction

Age-related deficits in memory for the temporal order of items or events in a sequence have been well documented in older humans (Newman et al., 2001; Kessels et al., 2007; Old and Naveh-Benjamin, 2008; Ulbrich et al., 2009; Blachstein et al., 2012; Tolentino et al., 2012; Roberts et al., 2014). Two recent studies demonstrated that temporal interference may play a key role in the ability of older adults to remember sequences of stimuli (Tolentino et al., 2012; Roberts et al., 2014). As reviewed by Kesner and Hopkins (2006), studies have shown that items occurring further apart in a temporal sequence are easier to remember than items that are temporally adjacent using various tests in both humans and animals. This is hypothesized to occur because there is more interference between temporally proximal stimuli in a sequence than temporally distant stimuli (Gilbert et al., 2001; Kesner and Hopkins, 2006; Tolentino et al., 2012; Roberts et al., 2014).

Tolentino et al. (2012) demonstrated that temporal order memory for fixed and random sequences of visuospatial stimuli was impaired in non-demented older adults compared to young adults, particularly when stimuli were close together in the sequence and temporal interference was high. A recent study by Roberts et al. (2014) tested young and older adults on a novel temporal discrimination test involving visually distinct objects with a wider range of temporal distances between stimuli relative to the study by Tolentino et al. (2012). Although there was individual variability among older adults, Roberts et al. (2014) reported that older adults were impaired relative to young adults on the temporal order task at a group level. One group of older adults was found to show a general impairment in temporal order memory compared to young adults when interference was moderate or low. However, the other group was impaired only when interference was moderate. Taken together, these studies demonstrate that the ability of older adults to remember sequences of stimuli may depend on the level of temporal interference.

Although age-related changes in temporal order memory have been studied rather extensively in older adults, little is known about temporal order memory abilities during middle age. A study by Stark et al. (2013) found that the ability to discriminate between highly similar visual objects begins to decrease in middle age. Based on this finding, it is possible that middle-aged adults also may experience difficulty when discriminating between stimuli close together in time due to increased interference. An impaired ability to remember sequences of stimuli and/or events when temporal interference is increased could have a significant impact of a variety of daily tasks. Therefore, the present study sought to examine the effects of interference on temporal order memory in young, middle-aged, and older adults. The present study tested two specific a priori hypotheses. First, it was hypothesized that when temporal interference is high, temporal order memory would be significantly better in young adults compared to both middle-aged and older adults. Second, it was hypothesized that when interference is low, temporal order memory would be significantly better in young and middle-aged adults compared to older adults. However, no differences were expected between young and middle-aged adults.

## Materials and Methods

### Participants

Participants consisted of 60 young adults 18–25 years of age, 43 middle-aged adults 40–55 years of age, and 43 non-demented older adults 65 years of age or older (demographic data summarized in Table 1). A subset of the data was included in previous publications (Tolentino et al., 2012; Nicoll et al., 2013; Woods et al., 2013). The average Dementia Rating Scale (Mattis, 1976) score for older adults was 135.52 (SE = 1.13) and all older adults scored above 123, providing evidence that none of the participants showed symptoms of dementia. A one-way analysis of variance (ANOVA) revealed a significant trend for years of education completed, *F*_(2,143)_ = 4.467, *p* = 0.06. As shown in Table 1, the older adults completed the highest years of education, followed by middle-aged adults. Therefore, age-related deficits in temporal order memory in middle-aged or older adults are not due to lower education levels in these groups. A chi-square analysis revealed a slight trend for gender difference among the three groups, *χ*^2^ (2, *N* = 146) = 4.94, *p* = 0.09. When gender or education was entered into the analyses discussed below, the variable was not significant in the model nor did the variable interact with age group; therefore, it was not included in the final analyses. All participants provided informed consent prior to participation approved by San Diego State University and the University of California, San Diego.

**Table 1:** **Mean (standard error of the mean) demographic variables for young adults (YA), middle-aged adults (MA), and older adults (OA)**.

	Young adults	Middle-aged adults	Older adults
**N** (total)	60	43	43
**Age** (yr)	20.8 (0.29)	48.70 (0.67)	75.26 (1.10)
**Gender** (% Female)	62%	40%	54%
**Education** (yr)	14.00 (0.30)	15.37 (0.37)	16.02 (0.54)

### Temporal Order Memory Test

Temporal order memory for random sequences of visuospatial stimuli was assessed using a well-characterized, computer-based task (Pirogovsky et al., 2009; Tolentino et al., 2012; Nicoll et al., 2013; Woods et al., 2013). Participants viewed a computerized radial eight-arm maze (approximately 30 cm in diameter) on a monitor. The maze consisted of eight arms extending from a center, similar to spokes on a wheel. Participants were told that a circle would appear at the end of each arm, one at a time, in a random order. The experimenter instructed the participants to remember the sequence in which the circles were presented on the arms.

Each trial consisted of a sample phase followed by a choice phase. During the sample phase, a gray circle (3-cm diameter) appeared randomly at the end of one of the eight arms on the maze. The circle appeared for 2 s, followed by a 2 s gray mask to eliminate after-image effects. Then another gray circle appeared at the end of a different, randomly selected arm for 2 s followed by another 2 s gray mask. This procedure continued until a circle was presented at the end of each of the eight arms once. The random sequence varied for each trial. Following the sample phase, the participant was presented simultaneously with two circles for 5 s, one at the end of one study phase arm and the other at the end of another study phase arm. The participant was asked to indicate which circle appeared earlier in the sequence. Temporal separation lags involving high (0 lag and 2 lag) and low (4 lag and 6 lag) levels of interference were randomly selected for each choice phase. The temporal lag represented the number of circles that appeared during the sample phase sequence between the two circles presented in the choice phase. For example, a 4 lag separation trial would consist of two choice phase circles that occurred with four circles between them during the sample phase sequence (e.g., 1st circle vs. 6th circle presented). Following each sample phase sequence, three choice phases were conducted involving three of the four temporal separations that were counterbalanced across sequences. Sixteen different sample phase sequences were presented with three choice phases for each sequence. There were a total of 12 choice phase trials for each of the four temporal separations. A 15 s inter-trial interval was implemented between each trial.

As reviewed above, it was hypothesized that there was more interference and a greater need to separate temporally proximal circles on 0 and 2 lag trials than temporally distant circles on 4 and 6 lag trials. In order to compare the performance of the three age groups on high interference and low interference trials, 0 and 2 lag trials were averaged and constituted the high interference condition and 4 and 6 lag trials were averaged to constitute the low interference condition.

### Statistical Analyses

ANOVA tests were conducted to examine the performance of young, middle-aged, and older adults on the high and low interference conditions. A Bonferroni correction was applied to control for multiple comparisons and the *p*-value for significance was set at *p* < 0.025. Tukey *post hoc* comparison tests were used to analyze group differences and effect size estimates were calculated using Cohen’s *d*.

## Results

**Figure 1:**
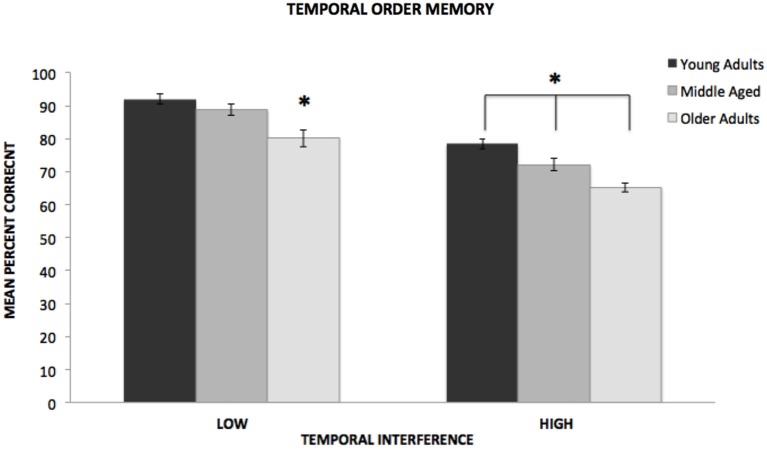
**Mean percent correct responses for young, middle-aged, and older adults on low and high temporal interference trials of the temporal order memory task**. *Indicates significance difference of *p* < 0.001.

In the low interference condition, the analysis revealed a significant difference among the age groups, *F*_(2,143)_ = 10.943, *p* < 0.001, *η*^2^ = 0.13. As shown in Figure 1, a *post hoc* Tukey test revealed that at low levels of interference, both the young (*p* < 0.05, *d* = 0.80) and middle-aged (*p* < 0.05, *d* = 0.59) groups significantly outperformed the older adult group. However, there was not a significant difference between the performance of the young and the middle-aged groups. In the high interference condition, the analysis also revealed a significant difference among the age groups, *F*_(2,143)_ = 14.497, *p* < 0.001, *η*^2^ = 0.17. As shown in Figure 1, a Tukey test revealed that the performance of all three groups differed at high levels of interference. The young group significantly outperformed both the middle-aged (*p* < 0.05,* d* = 0.53) and older groups (*p* < 0.05, *d* = 1.28). In addition, the middle-aged group significantly outperformed the older group (*p* < 0.05, *d* = 0.65).

## Discussion

The finding that older adults are impaired relative to young adults on a temporal order task is consistent with prior studies (Newman et al., 2001; Kessels et al., 2007; Old and Naveh-Benjamin, 2008; Ulbrich et al., 2009; Blachstein et al., 2012; Tolentino et al., 2012; Roberts et al., 2014). However, not all studies have found temporal order memory to be impaired in older adults (Perlmutter et al., 1981; Sekuler et al., 2006). As discussed in a recent paper published by Roberts et al. (2014), there are a number of differences between these studies that may have contributed to the discrepant findings (e.g., response times, exposure times, types of stimuli, temporal distance between pairs). The current findings also are consistent with recent studies by Tolentino et al. (2012) and Roberts et al. (2014), suggesting that temporal order memory abilities in older adults may be influenced by the level of interference involved in the task. The present findings demonstrate that older adults are impaired relative to young adults on a temporal order task when interference is high or low. However, the effect size associated with group differences between young and older adults was 62.5% higher in the high interference condition (*d* = 1.28) relative to the low interference condition (*d* = 0.80). This finding provides evidence that temporal order memory in older adults is poorer when interference is high.

The findings also provide novel insight into temporal order memory abilities during middle age. The data demonstrate that temporal order memory does not differ between middle-aged and young adults when temporal interference is low. However, deficits in temporal order memory can be detected in middle-aged adults, relative to young adults, when temporal interference is high using the current measure. The finding that middle-aged adults did not differ from young adults in the low interference condition, coupled with the greater effect associated with differences between young and older adults in the high interference condition compared to the low interference condition demonstrate that temporal order memory was differentially affected by interference levels. Taken together, these findings provide evidence that the observed age-related differences were not due solely to a general memory deficit.

Temporal order memory is thought to depend on frontal and temporal lobe function (Devito and Eichenbaum, 2011; Ekstrom et al., 2011). Humans with damage to the frontal (Milner et al., 1985; Shimamura et al., 1990; Daum and Mayes, 2000) or temporal lobes (Hopkins et al., 1995; Mayes et al., 2001; Spiers et al., 2001; Downes et al., 2002) show deficits on temporal order tasks. In addition functional neuroimaging studies have recorded activation in the frontal (Cabeza et al., 2000; Rowe and Passingham, 2001; Hayes et al., 2004; Knutson et al., 2004) and temporal lobes (Ekstrom and Bookheimer, 2007; Lehn et al., 2009) when humans perform tasks involving sequences of stimuli. Neuroimaging data indicate that the prefrontal cortex may be more involved in encoding of temporal information relative to the retrieval (Duarte et al., 2010). A recent study reported that cognitive decline during middle age may be mediated by gray matter changes in brain regions including the frontal lobes (Ferreira et al., 2014). Taken together, it is hypothesized that age-related changes in the frontal lobes of middle-aged adults may result in poorer encoding of temporally ordered stimuli resulting in impairments on the present task when stimuli are closer together in a sequence and temporal interference is high. However, when temporal interference is lessened and choice stimuli are further apart in the sequence, the encoding in middle-aged adults may be sufficient enough to make more accurate recency judgments.

Pattern separation is a mechanism for separating partially overlapping patterns of activation so that one pattern may be retrieved as separate from other patterns. A pattern separation mechanism may reduce interference among similar memory representations and increase the likelihood of accurate encoding and retrieval (Gilbert and Brushfield, 2009). The dentate gyrus (DG) and CA3 hippocampal subregions have been reported to support pattern separation (Kesner, 2007; Gilbert and Brushfield, 2009; Rolls, 2010; Yassa and Stark, 2011; Schmidt et al., 2012). Age-related changes in these subregions have been hypothesized to result in less efficient pattern separation due to strengthened processing of stored information at the expense of processing new information (Wilson et al., 2006; Yassa and Stark, 2011). Neurogenesis within the DG subregion also has been hypothesized to play a role in pattern separation (Aimone et al., 2011; Sahay et al., 2011; Luu et al., 2012) and there is evidence that the reductions in neurogenesis in old animals may be related to decreased hippocampal volume and impaired performance on hippocampal dependent tasks (Driscoll et al., 2006). One possible interpretation of the present findings is that pattern separation for temporal sequences is less efficient in middle-aged and older adults. Recent studies have hypothesized that pattern separation for temporal information may be impaired in older adults (Tolentino et al., 2012; Roberts et al., 2014). In the current study, the pattern separation demands are hypothesized to be greatest in the high interference condition, involving trials with the shortest temporal separation lags. Given that both middle-aged and older adults showed significant deficits relative to young adults in the high interference condition, the data provide evidence that less efficient pattern separation may contribute to age-related changes in temporal order memory. There is a growing body of literature providing evidence that older adults may be impaired relative to young adults on behavioral tasks hypothesized to tax pattern separation (Toner et al., 2009; Stark et al., 2010, 2013; Yassa et al., 2011a,b; Holden et al., 2012; Tolentino et al., 2012; Ly et al., 2013; Reagh et al., 2014; Roberts et al., 2014). In addition, a recent study by Stark et al. (2013) reported middle-aged adults were impaired on a behavioral pattern separation task involving visual objects. The present findings provide preliminary evidence that middle-aged adults also may show impairments consistent with a pattern separation deficit on a temporal order memory test.

Although future studies are needed with larger samples, the current findings offer unique insight into the effects of interference on temporal order memory during middle and old age. The findings show that age-related temporal order memory deficits may be detectable as early as middle age when a test is used that involves elevated levels of temporal interference. A recent study reported that impaired temporal order memory may be a selective behavioral marker of Alzheimer’s disease (Bellassen et al., 2012). In addition, temporal order memory deficits have been reported in older adults diagnosed with mild cognitive impairment (Gillis et al., 2013), which has been described as a transitional stage between normal aging and Alzheimer’s disease. Therefore, the present findings potentially may have both basic science and clinical implications.

## Conflict of Interest Statement

The authors declare that the research was conducted in the absence of any commercial or financial relationships that could be construed as a potential conflict of interest.
